# Occurrence of CYP2B6 516G>T polymorphism in patients with ARV‐associated hepatotoxicity

**DOI:** 10.1002/mgg3.598

**Published:** 2019-03-12

**Authors:** HariOm Singh, Sonam Lata, T. N. Dhole, Raman R. Gangakhedkar

**Affiliations:** ^1^ Department of Molecular Biology National AIDS Research Institute Pune India; ^2^ Department of Microbiology Sanjay Gandhi Post Graduate Institute of Medical Sciences Lucknow India; ^3^ Department of Clinical Sciences National AIDS Research Institute Pune India

**Keywords:** ARV‐associated hepatotoxicity, CYP2B6, genetic polymorphism, HIV patients, NNRTI drugs

## Abstract

**Background:**

Hepatic enzyme cytochrome P450 2B6 (CYP2B6) plays a role in the metabolism of efavirenz drugs. *CYP2B6* 516G>T variation showed an implication for HIV treatment.

**Methods:**

*CYP2B6 *516G>T polymorphism was genotyped in a total 165 HIV patients that include 34 with and 131 without hepatotoxicity and 155 healthy individuals by the PCR‐RFLP.

**Results:**

In patients with hepatotoxicity, the prevalence of *CYP2B6* 516TT genotype was higher as compared to healthy individuals (35.3% vs. 30.5%, OR = 1.74). Patients with hepatotoxicity using tobacco had a higher prevalence of genotypes *CYP2B6* 516GT, 516TT, 516GT+TT as compared to healthy individuals (28.57% vs. 25.93%; 57.14% vs. 29.63%; 85.71% vs. 55.56%). Likewise, hepatotoxicity in patients consuming alcohol showed higher distributions of *CYP2B6* 516GT, 516TT, 516GT+TT genotypes (57% vs. 25.93%; 42.86% vs. 33.33%; 71.43% vs. 59.26%). Nevirapine users with hepatotoxicity overrepresented genotypes *CYP2B6 TT *and 516GT+TT as compared to efavirenz users (47.83% vs. 45.45%, OR = 6.88, 65.22% vs. 54.55%, OR = 1.56). Similarly, in nevirapine +alcohol users with hepatotoxicity, the frequency of *CYP2B6 *516GT, 516GT+TT genotypes was higher than with nevirapine +alcohol nonusers (40.0% vs. 11.11%, OR = 8.00, 80.0% vs. 27.78%, OR = 4.00). In HIV patients, nevirapine users had higher frequency of *CYP2B6 *516GT, 516GT+TT genotypes as compared to efavirenz users (42.02% vs. 25.00%, OR = 2.53; 72.27% vs. 58.33%, OR = 1.86). Likewise, in HIV patients, genotypes *CYP2B6 *516GT, 516GT+TT were predominant with nevirapine +alcohol users as compared to nevirapine +alcohol nonusers (57.89% vs. 34.57%, OR = 2.46; 78.95% vs. 69.14%, OR = 1.67). In multivariate logistic regression, taking nevirapine had a protection for severity of ARV‐associated hepatotoxicity (OR = 0.23, *p* = 0.005).

**Conclusions:**

No significant association was detected between *CYP2B6* 516G>T polymorphism and susceptibility to ARV‐associated hepatotoxicity.

## 1 INTRODUCTION

Antiretroviral therapy (ART) has modified the natural history of HIV infection in both rich and limited‐resource countries (LRCs) like India. Two lines of treatment are widely available to treat the HIV patients and almost no test, aside from the CD4 cell count, is performed for monitoring treatment outcome. Long‐term efficacy and toxicity are major challenges when selecting an antiretroviral (ARV) regimen for treatment of AIDS. A common adverse drug reaction (ADR) is hepatotoxicity which leads to treatment interruptions in HIV patients (Dyke, Wang, & Williams, 2008). Individuals using nevirapine were associated with higher incidence of liver toxicity than efavirenz (van Leth et al., 2004). A 10.8% grade 3 or 4 hepatotoxicity was observed with efavirenz‐treated group and 8.9% in the nevirapine‐treated group (Reisler, Servoss, & Sherman, 2001). However, the incidence of hepatotoxicity due to nevirapine was 3.19% (Nagpal, Tayal, Kumar, & Gupta, 2010).

Mainly, drug metabolism and detoxification enzymes can be present in the liver and these are susceptible to injury. Single nucleotide polymorphism (SNP) in drug metabolizing enzyme genes may cause liver toxicity (Bissell, Gores, Laskin, & Hoofnagle, 2001). CYP2B6 (OMIM: 614,546) is an isoenzyme found on exon 4 of chromosome 19 and is characterized by wide interindividual and interethnic variation in expression and activity in the human liver in vitro* (*Saitoh et al., 2007). Drug metabolizing enzyme including CYP2B6 plays a major role in the hydroxylation of EFV and NVP (Erickson, Mather, Trager, Levy, & Keirns, 1999; Ward et al., 2003). A total of 29 alleles of *CYP2B6* (*1A [wild‐type] to *29) have been described (CYP2B6 allele nomenclature. www.cypalleles.ki.se/cyp2b6.htm 102), many of which are associated with increased, decreased, or abolished enzyme activity (Watanabe et al., 2010; Zanger et al., 2007; Zhang et al., 2011). CYP2B6*6 516G>T and 785A>G polymorphism were associated with “slow metabolism” of EFV (Zhang et al., (2011); Desta et al., 2007). The CYP2B6*6 516G>T gene polymorphism is accountable for aberrant splicing, resulting in a high‐splice variant and low* CYP2B6* expression phenotype (Hofmann et al., 2008). The *CYP2B6* 516 G>T gene polymorphism is associated with higher plasma EFV concentrations leading to increased drug‐related side effects (Haas et al., 2005; Nyakutira et al., 2008; Ribaudo et al., 2006; Rodriguez‐Novoa et al., 2005; Sadiq, Fredericks, Khoo, Rice, & Holt, 2005). A G>T change at position 516 of *CYP2B6* gene results in a Gln–His (Glutamine–Histidine) amino acid change which is associated with a significant reduction in CYP2B6 catalytic activity (Penzak et al., 2007). The 516TT genotype of *CYP2B6* is associated with reduced enzyme activity (Gatanaga et al., 2007).

Studies have reported an association between *CYP2B6* genotypes and the pharmacokinetics of EFV and NVP in HIV‐infected patients of European and African ethnicities (Cabrera et al., 2009; Haas et al., 2009; Mahungu et al., 2009; Motsinger et al., 2006; Ribaudo et al., 2007; Rotger et al., 2007; Schipani et al., 2011; Wyen et al., 2008). Also, similar studies have been published in other ethnically diverse patients (Carr, Porte, Pirmohamed, Owen, & Cortes, 2010; Chen et al., 2010; Lindfelt, O'Brien, Song, Patel, & Winslow, 2010; Puthanakit, Tanpaiboon, Aurpibul, Cressey, & Sirisanthana, 2009; Ramachandran et al.., 2009; To et al., 2009). In India, it is believed that we have different gene pools in south, north, east, and west regions. The frequency of *CYP2B6*516G>T polymorphism was reported only in South Indian population (Ramachandran et al., 2009). Hence, we evaluated the prevalence of *CYP2B6* 516G>T polymorphism in West Indian HIV patients.

## 2 MATERIAL AND METHODS

### 2.1 Subjects

This is a case–control study, undertaken between November 2012 and February 2015, at the outpatient clinics of National AIDS Research Institute, Pune. The study included 34 patients with hepatotoxicity (Grade III/IV) under NNRTI containing ART regimen, 131 HIV patients without hepatotoxicity confirmed by liver function test (LFT), and 155 age‐matched healthy controls. Patients with hepatotoxicity having hepatitis B, hepatitis C, tuberculosis, and concurrent untreated opportunistic infections, immune reconstitution syndrome and under any other known hepatotoxic drugs were excluded from cases. HIV patients having evidence of hepatotoxicity, hepatitis B, hepatitis C, tuberculosis, and receiving any other known hepatotoxic drugs were excluded. One hundred and fifty‐five individuals (those from the same family were excluded), HIV, Hepatitis B, C and Tuberculosis free, age‐matched and serum negative from HIV‐ELISA test were recruited. Clinical research proforma was filled to obtain clinical data by questionnaire, personal interviews, and review of case records. Liver function test was done to evaluate the status of liver enzyme. Total Bilirubin >3.22 mg/ml, SGOT >93.8 U/ml, SGPT >229.5 U/ml, and Alkaline phosphatase >550.8 U/ml for male hepatotoxicity cases and total Bilirubin >3.22 mg/ml, SGOT >163.2 U/ml, SGPT >173.4 U/ml, and Alkaline phosphatase >550.8 U/ml for female hepatotoxicity were considered as cases. Total Bilirubin <1.24 mg/ml, SGOT <32 U/ml, SGPT <34 U/ml, and Alkaline phosphatase <108 U/ml for male and female HIV‐infected control were considered. Estimation of CD4 count was done by fluorescence‐activated cell sorter (FACS). CD4 status was used to classify patients into different subgroups. CD4 ranges from <200 cells/mm^3^ were defined as an advanced stage, 201–350 cells/mm^3^ as an intermediate stage, and >350 cells/mm^3^ onward as an early stage. ELISA for hepatitis C and HBsAg testing was performed using Ortho HCV ELISA test system and Murex HBsAg confirmatory (Diasorin) ELISA. Environmental exposures such as tobacco and alcohol usage were also recorded in the questionnaire. The study was approved by the local institutional ethics committee and written informed consent was taken from all eligible participants.

## 3 DNA EXTRACTION

Two milliliters of peripheral blood sample was collected and stored at −70°C prior to DNA extraction. Genomic DNA extraction was done from peripheral blood leukocytes pellet using the AxyPrep Blood Genomic DNA Miniprep Kit according to the protocol given by the manufacturer.

### 3.1 Genotyping

The *CYP2B6 *(516G>T) was genotyped using PCR‐restriction fragment length polymorphism (PCR‐RFLP) in subjects participants. The amplification of *CYP2B6 *(516G>T) primers was taken as reported (Ramachandran et al., 2009). PCR was performed in a total volume of 20 μl with 20 pmol of each primer, genomic DNA (100–150 ng), 10 mmol/L deoxynucleotide triphosphates, PCR buffer containing 100 mmol/L Tris‐HCl, pH 8.6, 50 mmol/L KCl, 1.5 mmol/L MgCl_2_, and 1.5 units of Taq polymerase (Bangalore Genei, India). The reaction conditions for *CYP2B6 *516G>T were: initial denaturation at 94°C for 3 min, followed by 35 cycles of denaturation at 95°C for 30 s, annealing at 58°C for 30 s, extension at 72°C for 45 s, and final extension at 72°C for 7 min. Amplified product of *CYP2B6* was digested using restriction enzyme *BsrI* (Fermentas Inc.)*. *Genotyping of *CYP2B6 *was done in 15% polyacrylamide gel using molecular weight markers and visualized after staining with ethidium bromide. Based on sequences and location of SNP, genotypes of *CYP2B6* were as assigned as follows: for *CYP2B6: *204 bp for 516GG (good metabolizer); 152 and 52 bp for 516TT (poor metabolizer), and 204, 152, and 52 bp for 516GT (intermediate metabolizer) genotype. Veriti 96‐well Thermal cycler (Applied Biosystems) was used to amplify the desired DNA. PCR products and molecular weight markers were visualized after staining with ethidium bromide. Twenty percent of samples from both patients and controls were re‐genotyped by other laboratory personnel and no discrepancy in genotyping was noticed. Ten percent of samples were sequenced to assess the genotyping error.

### 3.2 Data analysis

The age variable was expressed as mean ± *SD*. The deviation from Hardy–Weinberg equilibrium in controls was analyzed using *χ* ^2^ goodness‐of‐fit test. We compared the genotype frequency between HIV patients with hepatotoxicity versus without hepatotoxicity and HIV patients versus healthy controls using *χ* ^2^ statistic (Fisher's exact test for cell size <5). Tobacco, alcohol usages, and genotypes interaction were examined in all eligible HIV patients. Odds ratios (ORs) and 95% confidence interval (CI) were calculated by unconditional binary logistic regression. All statistical analysis was performed using spss software version 17.0 (SPSS) and tests of statistical significance were two‐sided and taken as significant when *p‐*value was less than 0.05.

## 4 RESULTS

The study consisted of a total 165 HIV patients of which 34 patients had hepatotoxicity, 131 were without hepatotoxicity, and 155 were healthy controls. The mean age (years ±*SD*) of patients with hepatotoxicity, without hepatotoxicity, and healthy controls were 39.45 years ± 3.91, 38.39 years ± 4.23, and 36 years ±2.80, respectively. The demographic profile of HIV patients with, without hepatotoxicity, and healthy controls is shown in Table 1.

**Table 1:** Characteristics of HIV patients with hepatotoxicity, without hepatotoxicity, and healthy controls

S. no.	HIV patients with hepatotoxicity (Grade III and IV)	HIV patients without hepatotoxicity		Healthy controls
Number	*N* = 34	*N* = 131		*N* = 155
Mean age (range)	40.70 ± 7.63	39.41 ± 7.18		36.75 ± 8.50
Females	15 (44.11%)	47 (35.87%)		38 (25%)
Males	19 (55.88%)	84 (64.12%)		117 (75.48%)
NNRTI regimen				
Nevirapine *N* = 142	30 (88.23%)	112 (85.49%)		Not applicable (NA)
Efavirenz *N* = 23	4 (11.76%)	19 (14.50%)		NA
Alcohol habit				
User *N* = 51	23 (67.64%)	28 (21.37%)	13 (8.12%)	
Nonuser *N* = 114	11 (32.35%)	103 (78.62%)	21 (13.12%)	
Tobacco habit				
User *N* = 50	23 (67.64%)	27 (20.61%)	15 (9.37%)	
Nonuser *N* = 115	11 (32.35%)	104 (79.38%)	27 (16.87%)	
CD4+ status				
<200 (*N* = 95)	25 (73.52%)	61 (45.56%)	NA	
201–350 (*N* = 50)	9 (26.47%)	50 (38.16%)	NA	
>350 (*N* = 20)	0 (0)	20 (15.26%)	NA	

## 5 GENOTYPE–PHENOTYPE CORRELATION

### 5.1 ***CYP2B6*** **516G/T polymorphism and HIV patients with hepatotoxicity**

The genotype and allele frequency of *CYP2B6 *516G>T polymorphism in total HIV patients and healthy controls and HIV patients with and without hepatotoxicity are shown in Table 2. The distribution of *CYP2B6* 516 G>T polymorphism was not significantly different between total HIV patients and healthy controls. Higher distribution of *CYP2B6*516TT genotype was observed in HIV patients with hepatotoxicity as compared to without hepatotoxicity (35.3% vs. 30.5%, OR = 1.74, 95%CI: 0.66–4.60, *p* = 0.25). The *CYP2B6 *516GT genotype and 516T allele were not significantly different between both the groups (26.5% vs. 40.5% and 48.5%vs. 50.8%, respectively).

**Table 2:** Frequency distribution of *CYP2B6* 516G/T genotypes in a total HIV patients and healthy controls and HIV patients with and without hepatotoxicity

Genotypes *CYP2B6* 516 G/T	Total HIV patients *N* = 165 (%)	Healthy controls *N* = 155 (%)	*p*‐value	OR (95%CI)
GG	51 (30.90)	45 (29.0)	1	Reference
GT	62 (37.57)	68 (43.9)	0.50	0.80 (0.46–1.41)
TT	52 (31.51)	42 (27.1)	0.87	1.09 (0.59–2.01)
GT+TT	114 (69.09)	110 (71.0)	0.89	1.00 (0.58–1.73)

Alleles	Total HIV patients *N* = 330 (%)	Healthy controls *N* = 310 (%)	*p*‐value	OR (95%CI)
G	164 (49.69)	158 (51.0)	1	Reference
T	166 (50.30)	152 (49.0)	0.80	1.05 (0.76–1.45)

Genotypes *CYP2B6* 516 G/T	HIV patients with hepatotoxicity *N* = 34 (%)	HIV patients without hepatotoxicity *N* = 131 (%)	*p*‐value	OR (95%CI)
GG	13 (38.2)	38 (29.0)	1	Reference
GT	9 (26.5)	53 (40.5)	0.67	0.82 (0.32–2.07)
TT	12 (35.3)	40 (30.5)	0.25	1.74 (0.66–4.60)
GT+TT	21 (61.8)	93 (71)	0.40	0.66 (0.28–1.56)

Alleles	HIV patients with hepatotoxicity *N* = 68 (%)	HIV patients without hepatotoxicity *N* = 262 (%)	*p*‐value	OR (95%CI)
G	35 (51.5)	129 (49.2)	1	Reference
T	33 (48.5)	133 (50.8)	0.78	1.04 (0.80–1.35)

Note

*N* = number of subjects, (%) = frequency of genotypes/allele. Presence of GG for GT, TT, and GT+TT genotypes and G for T allele were taken as reference group for statistical analysis.

Chromosome 19 ‐ NC_000019.10.

### 5.2 ***CYP2B6*** **516G/T polymorphism and HIV patients**

The distribution of *CYP2B6 *516G>T polymorphism in HIV patients and healthy controls is presented in Table 3. *CYP2B6 *516G>T polymorphisms in healthy controls followed the Hardy–Weinberg equilibrium (*p* = 0.12). The *CYP2B6 *carriage 516TT genotype presented slightly higher in HIV patients as compared to healthy controls (30.5% vs. 27.1%, OR = 1.14, 95%CI: 0.63–2.06, *p* = 0.64). The difference in frequency of *CYP2B6 *516GT genotype and 516T allele was not statically significant between HIV patients and healthy controls (40.5% vs. 43.9% and 50.8% vs. 49.0%, respectively).

**Table 3:** Frequency distribution of *CYP2B6* 516 G/T genotypes in HIV patients and healthy controls

Genotypes *CYP2B6* 516 G/T	HIV patients *N* = 131(%)	Healthy controls *N* = 155 (%)	*p*‐value	OR (95%CI)
GG	38 (29.0%)	45 (29.0%)	1	Reference
GT	53 (40.5%)	68 (43.9%)	0.98	0.99 (0.52–1.87)
TT	40 (30.5%)	42 (27.1%)	0.64	1.14 (0.63–2.06)
GT+TT	93(71.0%)	110 (71.0%)	0.89	1.00 (0.58–1.73)

Alleles	HIV patients *N* = 262(%)	Healthy controls *N* = 310 (%)	*p*‐value	OR(95%CI)
G	129 (49.2%)	158 (51.0%)	1	Reference
T	133 (50.8%)	152 (49.0%)	0.68	0.93 (0.67–1.29)

Note

*N* = number of subjects, (%) = frequency of genotypes/allele. Presence of GG for GT, TT genotypes and G for T allele were taken as reference group for statistical analysis.

### 5.3 Genotype‐HIV disease stage correlation

*CYP2B6 *516G>T polymorphism was not significantly distributed between HIV disease stages and healthy controls. However, the presence of *CYP2B6*516TT genotype was greater in advanced HIV disease stages compared with healthy controls (30.38% vs. 27.1%, OR = 1.29, 95%CI: 0.58–2.84, *p* = 0.62) (Table 4).

**Table 4:** Frequency distribution of *CYP2B6* 516G/T genotypes in different HIV disease stages and healthy controls

Genotypes* CYP2B6 *516 G/T	Healthy controls *N* = 155 (%)	Early HIV disease stage		Intermediate HIV disease stage		Advanced HIV disease stage	
		*N* = 19 (%)	OR (P)	*N* = 33 (%)	OR (P)	*N* = 79 (%)	OR (P)
GG	45 (29.0%)	7 (36.84%)	1 (Reference	11 (33.34%)	1 (Reference	20 (25.32%)	1 (Reference)
GT	68 (43.9%)	4 (21.05%)	0.38 (0.22)	14 (42.42%)	0.84 (0.87)	35 (44.30%)	1.16 (0.79)
TT	42 (27.1%)	8 (42.11%)	1.22 (0.93)	8 (24.24%)	0.78 (0.81)	24 (30.38%)	1.29 (0.62)

Note

*N* = number of subjects, (%) = frequency of genotypes. Presence of GG for GT, TT genotypes were taken as reference group for statistical analysis.

### 5.4 Gene–environment interaction

In patients with hepatotoxicity along with tobacco usage genotypes *CYP2B6*516GT, 516TT and 516GT+TT genotypes were higher as compared to nonusers (28.57% vs. 25.93%, OR = 3.43, 95%CI: 0.18–117.72, *p* = 0.73; 57.14% vs. 29.63%, OR = 6.00, 95%CI: 0.45–170.81, *p* = 0.27; 85.71% 55.56%, OR = 4.80, 95%CI: 0.44–121.06, *p* = 0.30, respectively). Similarly, the frequency of distribution of genotype *CYP2B6*516GT was higher in HIV patients using tobacco as compared to nonusers (46.51% vs. 37.50%, OR = 1.31, 95%CI: 0.50–3.48, *p* = 0.70) (Table‐5).

The occurrence of *CYP2B6*516GT, 516TT, and 516GT+TT genotypes was higher in patients with hepatotoxicity using alcohol than in nonusers (28.57% vs. 25.93%, OR = 1.57, 95%CI: 0.12–21.71, *p* = 0.68; 42.86% vs. 33.33%, OR = 1.83, 95%CI: 0.18–20.78, *p* = 0.92; 71.43% 59.26%, OR = 1.72, 95%CI: 0.22–15.77, *p* = 0.87, respectively). Likewise, the occurrence of *CYP2B6*516GT and 516GT+TT genotypes was higher in HIV patients with alcohol users as compared to nonusers (54.54% vs. 33.33%, OR = 2.03, 95%CI: 0.77–5.43, *p* = 0.17; 74.99% vs. 68.97%, OR = 1.35, 95%CI: 0.55–3.33, *p* = 0.60, respectively) (Table 5)

**Table 5:** Frequency distribution of *CYP2B6* 516 G/T genotypes in HIV patients with and without hepatotoxicity using tobacco and alcohol

Genotypes *CYP2B6* 516 G/T	Tobacco user	Tobacco nonuser	*p*‐value	OR 95% CI
	*N* = 7 (%)	*N* = 27 (%)		
HIV patients with hepatotoxicity				
GG	1 (14.29%)	12 (44.44%)	1	Reference
GT	2 (28.57%)	7 (25.93%)	0.73	3.43 (0.18–117.72)
TT	4 (57.14%)	8 (29.63%)	0.27	6.00 (0.45–170.81)
GT+TT	6 (85.71%)	15 (55.56%)	0.30	4.80 (0.44–121.06)

Genotypes *CYP2B6* 516 G/T	Tobacco user *N* = 43 (%)	Tobacco nonuser *N* = 88 (%)	*p*‐value	OR 95% CI
HIV patients without hepatotoxicity				
GG	12 (27.91%)	26 (29.55%)	1	Reference
GT	20 (46.51%)	33 (37.50%)	0.70	1.31 (0.50–3.48)
TT	11 (25.58%)	29 (32.95%)	0.88	0.82 (0.28–2.43)
GT+TT	31 (72.09%)	62 (70.45%)	0.99	1.08 (0.45–2.63)

Genotypes *CYP2B6* 516 G/T	Alcohol user *N* = 7 (%)	Alcohol nonuser *N* = 27 (%)	*p*‐value	OR 95% CI
HIV patients with hepatotoxicity				
GG	2 (28.57%)	11 (40.74%)	1	Reference
GT	2 (28.57%)	7 (25.93%)	0.68	1.57 (0.12–21.71)
TT	3 (42.86%)	9 (33.33%)	0.92	1.83 (0.18–20.78)
GT+TT	5 (71.43%)	16 (59.26%)	0.87	1.72 (0.22–15.77)

Genotypes *CYP2B6* 516 G/T	Alcohol user *N* = 44 (%)	Alcohol nonuser *N* = 87 (%)	*p*‐value	OR 95% CI
HIV patients without hepatotoxicity				
GG	11 (25.00%)	27 (31.03%)	1	Reference
GT	24 (54.54%)	29 (33.33%)	0.17	2.03 (0.77–5.43)
TT	9 (20.45%)	31 (35.63%)	0.69	0.71 (0.23–2.22)
GT+TT	33 (74.99%)	60 (68.97%)	0.60	1.35 (0.55–3.33)

Note

*N* = number of subjects, (%) = frequency of genotypes. Presence of GG for GT, TT genotypes were taken as reference group for statistical analysis.

Table 6 shows the distribution of *CYP2B6 *516G>T polymorphism in patients with hepatotoxicity, without hepatotoxicity using nevirapine and efavirenz. In patients with hepatotoxicity, the distribution of *CYP2B6*516TT, 516GT+TT genotypes appeared to be higher in nevirapine users as compared to efavirenz users (47.83%vs. 9.09%, OR = 6.88, 95%CI: 0.55–189.71, *p* = 0.19; 65.22% vs. 54.55%, OR = 1.56, 95%CI: 0.29–8.64, *p* = 0.82, respectively). Similarly, *CYP2B6*516GT, 516TT, 516GT+TT genotypes were observed predominantly at a higher level in HIV patients with nevirapine use as compared to efavirenz use (42.02% vs. 25.00%, OR = 2.53, 95%CI: 0.48–14.49, *p* = 0.38; 30.25% vs. 33.33%, OR = 1.36, 95%CI: 0.28–6.74, *p* = 0.93; 72.27% vs. 58.33%, OR = 1.86, 95%CI: 0.47–7.17, *p* = 0.49, respectively).

**Table 6:** Frequency distribution of *CYP2B6* 516 G/T genotypes in NNRTI regimens using HIV patients with and without hepatotoxicity

Genotypes *CYP2B6* 516 G/T	Nevirapine user *N* = 23 (%)	Efavirenz user *N* = 11 (%)	*p*‐value	OR 95% CI
HIV patients with hepatotoxicity				
GG	8 (34.78%)	5 (45.45%)	1	Reference
GT	4 (17.39%)	5 (45.45%)	0.72	0.50 (0.06–3.82)
TT	11 (47.83%)	1 (9.09%)	0.19	6.88 (0.55–189.71)
GT+TT	15 (65.22%)	6 (54.55%)	0.82	1.56 (0.29–8.64)

Genotypes *CYP2B6* 516 G/T	Nevirapine user *N* = 119 (%)	Efavirenz user *N* = 12 (%)	*p*‐value	OR 95% CI
HIV patients without hepatotoxicity				
GG	33 (27.73%)	5 (41.67%)	1	Reference
GT	50 (42.02%)	3 (25.00%)	0.38	2.53 (0.48–14.49)
TT	36 (30.25%)	4 (33.33%)	0.93	1.36 (0.28–6.74)
GT+TT	86 (72.27%)	7 (58.33%)	0.49	1.86 (0.47–7.17)

Note

*N* = number of subjects, (%) = frequency of genotypes. Presence of GG for GT, TT and GT+TT genotypes were taken as reference group for statistical analysis.

In patients with hepatotoxicity *CYP2B6*516GT, 516TT, 516GT+TT genotypes were found to be much prevalent in nevirapine +alcohol users as compared to nevirapine +alcohol nonusers (40.0% vs. 11.11%, OR = 8.00, 95%CI: 0.28–449.98, *p* = 0.41; 40.0% vs. 44.44%, OR = 2.00, 95%CI: 0.10–69.78, *p* = 0.92;80.0% vs. 27.78%, OR = 4.00, 95%CI: 0.31–110.70, *p* = 0.46, respectively). In HIV patients, *CYP2B6*516GT, 516GT+TT genotypes were distributed at a higher level with nevirapine +alcohol users in comparison to nevirapine +alcohol nonusers (57.89% vs. 34.57%, OR = 2.46, 95%CI: 0.84–7.31, *p* = 0.10; 78.95% vs. 69.14%, OR = 1.67, 95%CI: 0.62–4.62, *p* = 0.37, respectively) (Table 7).

**Table 7:** Frequency distribution of *CYP2B6* 516 G/T genotypes in combined NNRTI regimen using HIV patients with and without hepatotoxicity

Genotypes *CYP2B6* 516 G/T	Nevirapine + alcohol user *N* = 5 (%)	Nevirapine + alcohol nonuser *N* = 18 (%)	*p*‐value	OR 95% CI
HIV patients with hepatotoxicity				
GG	1 (20.0%)	8 (44.44%)	1	Reference
GT	2 (40.0%)	2 (11.11%)	0.41	8.00(0.28–449.98)
TT	2 (40.0%)	8 (44.44%)	0.92	2.00(0.10–69.78)
GT+TT	5 (80.0%)	10 (27.78%)	0.46	4.00(0.31–110.70)

Genotypes *CYP2B6* 516 G/T	Efavirenz + alcohol user *N* = 2 (%)	Efavirenz + alcohol nonuser *N* = 9 (%)	*p*‐value	OR 95% CI
GG	2 (100%)	3 (33.33%)	1	Reference
GT	0	5 (55.56%)	NC	–
TT	0	1 (11.11%)	NC	–
GT+TT	0	6 (66.67%)	NC	–

Genotypes *CYP2B6* 516 G/T	Nevirapine + alcohol user *N* = 38 (%)	Nevirapine + alcohol nonuser *N* = 81 (%)	*p*‐value	OR 95% CI
HIV patients without hepatotoxicity				
GG	8 (21.05%)	25 (30.86%)	1	Reference
GT	22 (57.89%)	28 (34.57%)	0.10	2.46 (0.84–7.31)
TT	8 (21.05%)	28 (34.57%)	0.10	0.89 (0.25–3.13)
GT+TT	30 (78.95%)	56 (69.14%)	0.37	1.67 (0.62–4.62)

Genotypes *CYP2B6* 516 G/T	Efavirenz + alcohol user *N* = 6 (%)	Efavirenz + alcohol nonuser *N* = 6 (%)	*p*‐value	OR 95% CI
GG	3 (50.0%)	2(33.33%)	1	Reference
GT	2 (33.33%)	1 (16.67%)	0.57	1.33 (0.03–76.89)
TT	1 (16.67%)	3 (50.0%)	0.70	0.20 (0.00–7.34)
GT+TT	3 (50.0%)	4 (66.67%)	1.00	0.50 (0.02–8.76)

Note

*N* = number of subjects, (%) = frequency of genotypes. Presence of GG for GT, TT and GT+TT genotypes were taken as reference group for statistical analysis. NC = not calculable.

### 5.5 Risk factors of ARV‐associated hepatotoxicity: Multivariate logistic regression analysis

Correlation of age, sex, tobacco, alcohol, baseline CD4 counts, and *CYP2B6* 516G/T polymorphism with ARV‐associated hepatotoxicity were done by multivariate logistic regression analysis. *CYP2B6* 516G/T polymorphism, age, sex, tobacco, alcohol usage, and baseline CD4 counts were not independent risk factors for ARV‐associated hepatotoxicity. While comparing between HIV patients with and without hepatotoxicity, *CYP2B6* 516TT genotype, taking alcohol and male HIV patients with hepatotoxicity showed a risk for ARV‐associated hepatotoxicity (OR = 1.79, *p* = 0.27; OR = 1.91, *p* = 0.63; OR = 1.90, *p* = 0.16, respectively)  (Table 8).

**Table 8:** Multivariate analysis between HIV patients with and without hepatotoxicity

Variables	B	S.E.	*df*	*p*‐value	OR (95%CI)
516GG			2	0.45	
516GT	−0.001	0.516	1	0.99	0.99 (0.36–2.74)
516 TT	−0.586	0.540	1	0.27	1.79 (0.62–5.17)
Age	−0.026	0.032	1	0.41	0.97 (0.91–1.03)
Sex	0.644	0.465	1	0.16	1.90 (0.76–4.73)
Tobacco user	0.196	0.608	1	0.747	1.217 (0.370–4.003)
Alcohol user	0.647	0.637	1	0.637	1.91 (0.766–4.739)
NNRTI drug user	−1.455	0.517	1	**0.005**	0.233 (0.085**–**0.643)
Baseline CD4 count	0.004	0.001	1	**0.001**	1.004 (1.002–1.007)

Note

*CYP2B6* 516G/T polymorphism, Age 18–50 year, sex, tobacco user, alcohol user, NNRTI drug user, Baseline CD4. Significant values (<0.05) represented in bold.

## 6 DISCUSSION

The differences in the activity of drug metabolizing enzyme contribute to interpatient variability in response to drugs. The SNPs of CYP450 enzymes are known to have a role in influencing the efficacy of therapy (Servoss et al., 2006). The SNPs within the *CYP2B6* gene are associated with altered hepatic *CYP2B6* expression and activity (Tong et al., 2006). The interindividual differences in hepatic *CYP2B6* expression and enzymatic activity may result in variable systemic exposure and therapeutic response to the drugs metabolized by *CYP2B6*.

The *CYP2B6* gene is highly polymorphic (Lang et al., 2001) and encodes the major enzymes for hepatic elimination of efavirenz and nevirapine. The prevalence of T allele varies within the populations (Tong et al., 2006; Xu et al., 2007). The variant 516 T allelic is more common in African‐Americans (20%) than in Hispanics (6.7%), or Caucasians (3.4%) and has been reproducibly associated with slower clearance and ethnic difference in efavirenz plasma concentrations (Haas et al., 2009). Ethnicity plays a key role, as reflected by the variation in frequency of T allele across the populations. In this study, the distribution of *CYP2B6* 516 G>T polymorphism was not significantly different among HIV patients and healthy controls, patients with hepatotoxicity versus without hepatotoxicity. In our study in the West Indian, a prevalence of 30.5% was observed for 516TT genotype in healthy controls as compared to 42% represented in South India (Servoss et al., 2006). The distribution of T allele frequency was 16% in the Japanese (Hiratsuka et al., 2002), 14% in Koreans (Cho et al., 2004), 42% in West Africans, 62% in Papua New Guineans (Mehlotra et al.., 2011), 28% in white Americans, 25% Caucasians (Haas et al., 2009), 28% African‐Americans (Haas et al., 2009), 43% and 34.5% Chinese (Tong et al., 2006; Xu et al., 2007), 43% Hispanics (Haas et al., 2009), 42% South India (Servoss et al., 2006), and 49.0% in this study.

In this is a case–control study, the current CD4 count was considered as a substitute of current HIV disease stage. As the time points for HIV infection were not known, the results may be confounded by the duration of HIV infection. In our study, the distribution of *CYP2B6 *516G/T polymorphism was not significantly different between HIV disease stages and healthy controls.

The gene–environment interactions determine the pathophysiology of the disease (Deng, Newman, Dunne, Silburn, & Mellick, 2004). However, for case–control association studies for environmental influences, cases must have matched controls (Greenland, 1980). It is assumed that a case study is always better to look at for the effect of gene–environment. Here, we have chosen the case‐only analysis. Heavy alcohol consumption had a negative impact on the CD4 cell counts of HIV patients naïve to ART (Samet et al., 2007). In our study, *CYP2B6*516TT genotype modulated the severity risk in patients with hepatotoxicity using tobacco (OR = 6.00, *p* = 0.27). Hepatotoxicity risk was revealed among alcohol users, with *CYP2B6*516TT genotype (OR = 1.83, *p* = 0.92). Similarly, presence of *CYP2B6 *516GT genotype among alcohol users resulted in the modulation of risk for acquisition of ARV‐associated hepatotoxicity (OR = 2.03, *p* = 0.17).

*CYP2B6 *516TT genotype also indicated a risk for hepatotoxicity severity in nevirapine users (OR=6.88, *p* = 0.19). Likewise, *CYP2B6 *516TT genotype among combined nevirapine +alcohol users has shown a risk for hepatotoxicity severity (OR = 2.00, *p* = 0.92). *CYP2B6 *516GT+TT combined genotype revealed a risk for acquisition of ARV‐associated hepatotoxicity and its severity in combined alcohol and nevirapine users (OR = 1.67, *p* = 0.37; OR = 4.00, *p* = 0.46, respectively). In multivariate logistic regression, while comparing between HIV patients with and without hepatotoxicity, *CYP2B6* 516TT genotype showed a risk for ARV‐associated hepatotoxicity (OR = 1.79, *p* = 0.27).It supports the idea that individuals with *CYP2B6*516TT genotype and alcohol and nevirapine usage are more prone to alcohol and drug‐related hepatotoxicity.

Limitations of this study are as follows: (a) it could only define the association; (b) could not determine the causality; (c) could not estimate the plasma efavirenz and nevirapine level in our subjects; (d) We had distributed a ratio of 1:4 for case controls. However, we could not complete matched enrollment in controls although our case–control ratio is around 1:3.

## 7 CONCLUSION

The distribution of *CYP2B6 *516G>T polymorphism was not significantly different among HIV patients and healthy controls though, the prevalence of *CYP2B6 *516TT genotype was higher in patients with hepatotoxicity. *CYP2B6* genotype along with nevirapine and alcohol usage may influence the acquisition of ARV‐associated hepatotoxicity and hepatotoxicity severity. The prevalence of *CYP2B6 *516TT genotype varied among different ethnic groups. Hence, further study of correlation of gene polymorphism with drug levels should be done in HIV patients on efavirenz 400 mg in a larger sample size. Finding of study may help to predict the acquisition of hepatotoxicity and also may influence the choice of drug to maintain the doses and its responses.

## CONFLICT OF INTEREST

None declared.
